# Management of Lateral Epicondylitis: A Prospective Comparative Study Comparing the Local Infiltrations of Leucocyte Enriched Platelet-Rich Plasma (L-aPRP), Glucocorticoid and Normal Saline

**DOI:** 10.5704/MOJ.2203.009

**Published:** 2022-03

**Authors:** KK Arora, R Kapila, S Kapila, A Patra, P Chaudhary, A Singal

**Affiliations:** 1 Department of Orthopaedics, Government Medical College, Amritsar, India; 2 Department of Oral Surgery, Sri Guru Ram Das Institute of Dental Sciences and Research, Amritsar, India; 3 Department of Anatomy, All India Institute of Medical Sciences, Bathinda, India

**Keywords:** corticosteroid, lateral epicondylitis, leucocyte-activated platelet-rich plasma, tennis elbow

## Abstract

**Introduction:**

Lateral epicondylitis is a painful condition of the elbow, characterised by pain and tenderness with resisted wrist extension. This study was carried out to evaluate the comparative efficacy of the local infiltration of L-PRP, methylprednisolone and normal saline in patients with lateral epicondylitis.

**Materials and methods:**

Sixty adult patients, between the ages 30 to 50 years, diagnosed with lateral epicondylitis of more than 12 weeks, were enrolled in the prospective randomised study. Their medical history and previous conservative treatment were recorded; the clinical evaluation of the tendinitis was made with the visual analogue scale (VAS), the disabilities of the arm, shoulder, and hand (DASH) outcome scores, the modified elbow performance index (MEPS), the functional assessment by patient-rated tennis elbow evaluation (PRTEE), together with the laboratory investigations. The patients were randomised using the computer-generated alphabets into three groups of 20: group A received saline, group B received PRP, and group C received corticosteroids.

**Results:**

Patients were seen at 4, 8 and 12 weeks to evaluate the post-injection status. VAS, DASH, and PRTEE scores were significantly reduced, and MEPS was significantly improved in group B compared to group A and group C. Moreover, the reductions in VAS and PRTEE were significantly different in group C compared to group A.

**Conclusion:**

PRP leads to superior healing with long-term therapeutic advantages compared to corticosteroids though it takes a little longer to have its effect.

## Introduction

Lateral epicondylitis (tennis elbow) is the commonest, chronic, disabling, painful condition of the elbow with an incidence rate of 1% to 3%, in adults between 35 and 50 years of age, having an equal male to female sex ratio, and manifesting itself on the resisted extension of the wrist joint. In India, it is frequently seen in people whose occupation requires frequent rotary motion of the forearm: carpenter, gardener, computer, and knitting workers. The dominant upper limb is mainly affected.

First described in 1873, it often is non-traumatic, with sharp contained pain at the lateral epicondyle, aggravating with grasping and rotatory movements, and wrist palmar flexion. In the past, it was thought to be due to an inflammation of the common extensor origin of the forearm^1^. However, it has been disproved histopathologically, and the term 'epicondylitis' itself is a misnomer^2-4^. Instead, it is a form of tendinosis resulting from repetitive stress-mediated degeneration of the common extensor tendon origin^5,6^.

Researchers have now proposed a pathophysiological integrative model which hypothesises an integration of local tendon pathology, changes in the pain system and impairment in the motor system as causal factors behind the origin of tennis elbow^7^.

Clinically there is maximum point tenderness, 5mm anterior, and just distal to the lateral epicondyle at the common extensor tendon origin, comprising the extensor carpi radialis brevis (ECRB) and extensor digitorum communis (EDC) muscles with decreased grip strength, limited supination, and dorsiflexion of the wrist^2^.

Multiple treatment interventions, including physical therapy, corticosteroid injections, NSAIDs, bracing, and acupuncture, as well as open and arthroscopic surgical debridement, have been advocated for it^1-3^.

Activated leucocyte enriched platelet-rich plasma (L-aPRP) is a promising innovative treatment option^8-14^. Leucocyte loaded, platelet-rich plasma, activated with thrombin, delivers various growth factors to the injury site^15-18^. A high concentration of these growth factors repairs tendon and ligament injury, thus quickening the tendon healing process^16,21^. During this healing process, tendons are much more receptive to circulation-derived/locally produced growth factors, most of which originate within the PRP^22,23-27^.

In this study, the comparative efficacy of a single administration of locally infiltrated leucocyte enriched, activated platelet-rich plasma (L-aPRP), glucocorticoid or normal saline, each as a treatment modality for lateral epicondylitis, were prospectively studied.

## Materials and Methods

This was a prospective comparative study of 60 patients of either sex, having lateral epicondylitis, from May 2018 to April 2019 at a tertiary institute of Punjab, India, to compare the efficacy of locally infiltrated leucocyte enriched, activated platelet-rich plasma (L-aPRP) to glucocorticoid and to normal saline single shot infiltration, as a treatment modality for lateral epicondylitis. This study was done on the outpatient department (OPD) patients who did not respond to other conservative treatment methods for lateral epicondylitis like non-steroidal anti-inflammatory drugs (NSAIDs), physiotherapy, tennis elbow support application and/or changing the nature of their job. After obtaining verbal and written consent for their inclusion into the study, the procedure was explained. In addition, prior approval of the institutional ethical committee (IEC) was also obtained.

Three groups of 20 patients each, selected by an allocation through computer-generated alphabeticals, for each method of infiltration and were named as group A, B, and C to assess each drug/normal saline (to note its placebo effect if any) infiltrated locally.

Inclusion criteria: (1) patients aged between 30-50 years, of either sex, (2) pain due to one-sided lateral epicondylitis that persisted for at least 12 weeks, (3) tenderness on pressure limited to regions around the elbow joint, (4) Complaints of pain during resisted extension of the middle finger or the wrist (Maudsley’s test), (5) positive Cozen's test, Thomson's test and/or Mill's Test.

Exclusion criteria: (1) patients with age <30 years, (2) diabetes mellitus (uncontrolled blood glucose >180mg% even with anti-diabetic drugs), (3) cervical radiculopathy, (4) rheumatoid arthritis, (5) pregnancy, (6) haemoglobin <10 mg/dl, (7) platelet count <150,000/mm^3^, (8) patients on aspirin, or similar anticoagulant drugs, (9) fibromyalgia, (10) pain in hand or shoulder or neck in the same upper limb, (11) ulcers over the elbow, (12) steroid injection within the last three months, (13) tumours in the upper limb.

Infiltration of a single dose of 3cc normal saline (0.9%) for group A patients, 3cc freshly prepared autologous leucocyte enriched activated platelet-rich plasma (L-aPRP) for group B patients, and 1ml (40mg) of methylprednisolone in 2ml of (1%) 10mg/ml lignocaine for group C patients, was administered in the outpatient department (OPD).

The leucocyte enriched, activated platelet-rich plasma (L-aPRP) was prepared using desktop size, a 9001-2000 ISO certified R-23 centrifuge apparatus.

Autologous leucocyte enriched activated platelet-rich plasma (L-aPRP, 1000000 platelets per microlitre of blood with leucocytes) was obtained from freshly drawn 30cc of venous blood with 22 G needle using 50cc disposable syringe, from the patient with an added anticoagulant (sodium citrate). The collected blood, under sterile conditions, was subjected to two sets of centrifugations (spins)^28^. The first spin, known as HARD SPIN (more than 3000 rpm for 15 minutes), separated the red blood cells (RBC) from the plasma containing the platelets, leucocytes, and clotting factors. Three layers resulted from the hard spin: an upper layer containing platelets and leucocytes, a middle layer known as the Buffy coat containing only leucocytes, and a bottom layer containing red blood cells (RBC). This bottom layer of red blood cells was separated and discarded.

The second spin, called SOFT SPIN (more than 2000 rpm for 5 minutes), separated the leucocyte enriched platelet-rich plasma (L-PRP) in the bottom of the tube from the platelet-poor plasma (PPP) at the top of the tube by the removal of more red blood cells and creating a bottom layer rich in platelets and leucocytes^29^. The bottom layer was further activated with thrombin. This leucocyte enriched, activated platelet-rich plasma (L-aPRP) was used for infiltration in group B patients.

Plain radiographs in two views of the affected elbow were done to exclude any bony pathology. Ultrasound and magnetic resonance imaging (MRI) confirmed the presence and extent of tendon injury. Before infiltration, pain and elbow function were assessed using four different measuring scores.

**Fig. 1:**
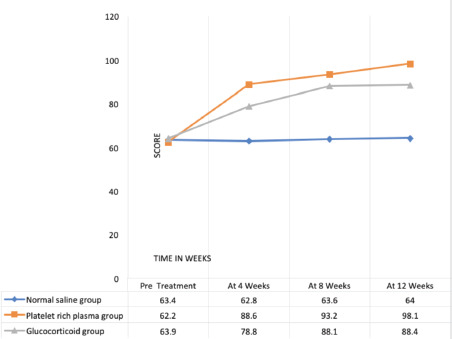
Comparison of mayo elbow performance score (MEPS), (pre-treatment and post infiltration of normal saline, L-PRP, Glucocorticoid at follow-up visits of 4, 8, and 12 weeks).

The Mayo elbow performance score (MEPS) (Table I, Fig. 1) reflected the elbow function of the patient and incorporated pain, movement, stability and activity of daily living. Out of a total score of 100 (100, the best one and 0, the worst one), the pain had 45 points, movement (range and arc of motion) 20, and stability 10, while daily functioning activities had 25 points^30^.

**Table I:** Mayo elbow performance score (MEPS)

Function	Point Score
Pain (45 Points)	
None	45
Mild	30
Moderate	15
Severe	0
Motion (20 Points)	
Arc 100°	20
Arc 50° to 100°	15
Arc 2°	5
Stability (10 Points)	
Stable	10
Moderate Instability	
Gross Instability	0
Daily Functions (25 points)	
Combing Hair	5
Feeding Oneself	5
Hygiene	5
Putting On Shirt	5
Putting On Shoes	5
Maximum Possible Total 100	

**Fig. 2:**
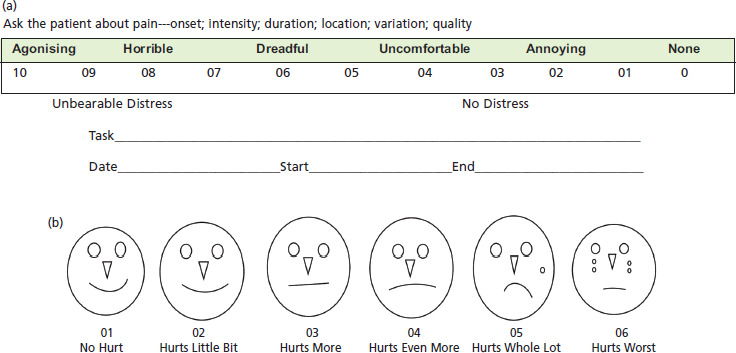
(a) VAS scale: numerical description for pain of lateral epicondylitis. (b) VAS scale: pictorial facial presentation for pain of lateral epicondylitis.

**Fig. 3:**
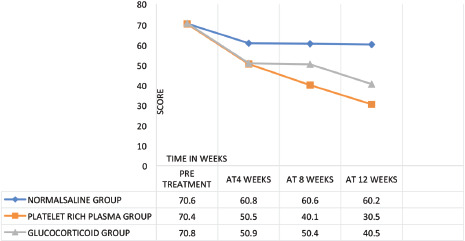
Comparison of VAS score, (pre -treatment and post infiltration of normal saline, l-PRP, glucocorticoid at follow-up visits of 4, 8, and 12 weeks).

Visual Analogue Scale (VAS) (Fig. 2 and 3) measured a characteristic or attitude of pain noted by the patients. Scores ranged from 0 (no pain) to 100 (severest pain). The Visual Analogue Scale score recorded by measurement in millimetres from the right-side end of the line up to the point that the patient marked. The outcome was measured by the changes in pain at pre-injection and subsequently at four, eight and 12 weeks^31^.

**Fig. 4:**
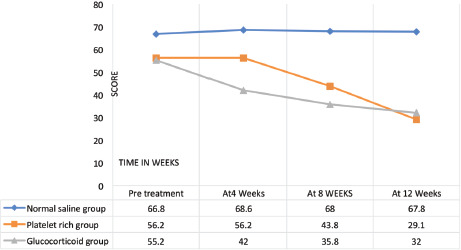
Comparison of DASH score, (pre -treatment and post infiltration of normal saline, l-PRP, glucocorticoid at follow-up visits of 4, 8, and 12 weeks).

The Disabilities of the Arm, Shoulder and Hand score (DASH) (Table II, Fig. 4) had 30 items with self-report questionnaires structured to assess physical activity and symptoms. The scores for 30 items are taken to calculate a total score ranging from 0 (no disability) to 100 (severest disability). A minimum of 27 of the 30 items must be completed for a score to be calculated^32^.

**Table II:** DASH score for shoulder, elbow and hand

No	Activity	Difficulty				
1	Open a tight jar or new jar	No difficulty	Mildly difficult	Moderately difficult	Severely difficult	Unable
2	Write	No difficulty	Mildly difficult	Moderately difficult	Severely difficult	Unable
3	Turn a key	No difficulty	Mildly difficult	Moderately difficult	Severely difficult	Unable
4	Prepare a meal	No difficulty	Mildly difficult	Moderately difficult	Severely difficult	Unable
5	Push open a heavy door	No difficulty	Mildly difficult	Moderately difficult	Severely difficult	Unable
6	Place an object on a shelf above the level of head	No difficulty	Mildly difficult	Moderately difficult	Severely difficult	Unable
7	Do heavy household jobs	No difficulty	Mildly difficult	Moderately difficult	Severely difficult	Unable
8	Garden or yard work	No difficulty	Mildly difficult	Moderately difficult	Severely difficult	Unable
9	Make a bed	No difficulty	Mildly difficult	Moderately difficult	Severely difficult	Unable
10	Carry a shopping bag or briefcase	No difficulty	Mildly difficult	Moderately difficult	Severely difficult	Unable
11	Carry a heavy object	No difficulty	Mildly difficult	Moderately difficult	Severely difficult	Unable
12	Change a light bulb overhead	No difficulty	Mildly	Moderately	Severely difficult	Unable
13	Wash or blow dry your hair	No difficulty	Mildly difficult	Moderately difficult	Severely difficult	Unable
14	Wash your back	No difficulty	Mildly difficult	Moderately difficult	Severely difficult	Unable
15	Put on a pullover sweater	No difficulty	Mildly difficult	Moderately difficult	Severely difficult	Unable
16	Use a knife to cut food	No difficulty	Mildly difficult	Moderately difficult	Severely difficult	Unable
17	Recreational activities which require little effort (eg. knitting, card playing)	No difficulty	Mildly difficult	Moderately difficult	Severely difficult	Unable
18	Recreational activities in which you take some forces or impacts through your arm, shoulder, or hand (eg. hammering, tennis, etc)	No difficulty	Mildly difficult	Moderately difficult	Severely difficult	Unable
19	Recreational activities in which you move your arm freely (eg. playing badminton)	No difficulty	Mildly difficult	Moderately difficult	Severely difficult	Unable
20	Manage transposition needs (getting one place to another)	No difficulty	Mildly difficult	Moderately difficult	Severely difficult	Unable
21	Sexual activities	No difficulty	Mild difficulty	Moderately difficult	Severely difficult	Unable
22	During the past week, to what extent your arm, shoulder or hand problem interfered with your normal social activities with family, friends, neighbors?	Not at all	Slightly	Moderately	Quite a bit	Unable
23	During past week were you limited in your work as a result of your arm, shoulder or hand problem?	Not limited	Slightly limited	Moderately limited	Very limited	Unable
24	Arm, shoulder, or hand pain	None	Mild	Moderate	Severe	Extreme
25	Arm, shoulder, or hand pain when you performed any specific activity?	None	Mild	Moderate	Severe	Extreme
26	Tingling (pins and needles) in your arm, shoulder, or hand	None	Mild	Moderate	Severe	Extreme
27	Weakness in your arm, shoulder, or hand	None	Mild	Moderate	Severe	Extreme
28	Stiffness in arm, shoulder, or hand	None	Mild	Moderate	Severe	Extreme
29	Last week how much difficulty have you had sleeping because of pain	No difficulty	Mildly difficult	Moderately difficult	Severely difficult	Can’t sleep

**Fig. 5:**
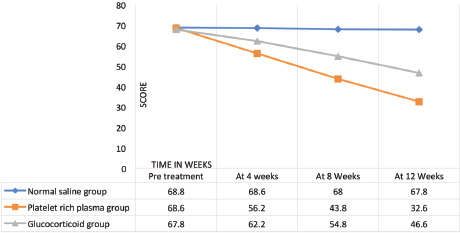
Comparison of PRTEE score, (pre-treatment and post infiltration of normal saline, l-PRP, glucocorticoid at follow-up visits of 4, 8, and 12 weeks).

Patient-rated tennis elbow evaluation (PRTEE) (Table III, Fig. 5) was for the functional assessment of the elbow joint. It was a 15-item questionnaire designed to measure forearm pain and disability in patients with LE. The PRTEE consisted of two subscales: the pain subscale and the function subscale; the best score was zero, and the worst score was 100. Thus, a total score was the sum of both pain and function^33^.

**Table III:** PRTEE – patient rated tennis elbow evaluation

Item	0	1	2	3	4	5	6	7	8	9	10
Pain											
Pain - When it is at its worst	0	1	2	3	4	5	6	7	8	9	10
Pain - At rest	0	1	2	3	4	5	6	7	8	9	10
Pain - When lifting a heavy object	0	1	1	2	3	4	5	5	6	6	7
Pain - When doing a task with repeated elbow movement	0	1	2	3	4	4	5	5	6	6	7
How often do you have pain?	0	1	2	3	4	4	5	5	6	6	7
Specific Activities											
Comb my hair	0	1	1	1	2	2	2	3	3	3	4
Eat with a fork or spoon	0	1	1	1	2	2	3	3	3	3	4
Pull a heavy object	0	1	1	1	2	2	2	3	3	3	4
Use my arm to rise from a chair	0	1	1	2	2	3	3	4	4	4	5
Carry a 10lb object with my arm at my side	0	1	2	2	3	3	4	4	4	4	5
Throw a small object, such as a tennis ball	0	1	1	2	2	2	3	3	3	3	4
Use a telephone	0	1	1	2	2	3	3	3	4	4	5
Do up buttons on the front of my shirt	0	1	1	2	2	3	3	3	4	4	5
Wash my opposite armpit	0	1	1	1	2	2	2	3	3	3	4
Tie my shoe	0	1	1	1	1	2	2	2	2	2	3
Turn the doorknob and open a doora	0	1	1	2	2	2	2	3	3	3	4
Usual Activities											
Personal activities (dressing, washing)	0	1	1	2	2	3	3	4	5	6	7
Household work (cleaning, maintenance)	0	1	2	3	4	5	6	7	8	9	10
Work (your job or everyday work)	0	1	1	2	2	3	3	4	4	4	5
Recreational activities	0	1	1	2	2	3	3	3	4	4	5

All infiltrations were done under sterile conditions using a 22-gauge needle locally directly over the centre of the lateral epicondyle, perpendicular to the skin (if the patient had sufficient subcutaneous fat) or at a 45° angle to a depth of 0.75cm to 1.5cm. The patient was kept in a supine position for 15 minutes after the infiltration and then sent home with instructions to restrict the use of the arm and elbow for the next 24 hours.

Post infiltration scores were re-evaluated, using the same questionnaires used pre-infiltration to evaluate the efficacy of one treatment modality over the other in the management of lateral epicondylitis.

## Results

In this study, the middle aged (30-50 years) group was commonly involved, especially as the skilled manual workers without any significant gender bias.

Most of the patients opted for the local infiltrations, as there was no improvement in their signs/symptoms with other conservative methods.

Post infiltration, the patients were followed-up in the orthopaedics outpatient department at the 3rd, 6th, and 12th weeks for assessment of clinical improvement in signs/symptoms of lateral epicondylitis.

Functionality parameters remained unchanged from their pre-infiltration status at the elbow joint with normal saline infiltrations when measured with MEPS, VAS, DASH, and PRTEE.

Leucocyte enriched activated platelet-rich plasma (L-aPRP) infiltrations had a continuous progressive, positive effect on the healing process, with a significant decrease of VAS, DASH, PRTEE scores and a significant rise MEPS score.

Glucocorticoid infiltrations decreased the severity of pain and increased MEPS, DASH, and PRTEE functionality due to anti-inflammatory action (Fig. 1 - 4), yet those effects were short-lived and stopped improving further after a few weeks. In addition, a few (n=4) patients reported hypopigmentation at the infiltration site.

Glucocorticoid and leucocyte enriched activated platelet-rich plasma (L-aPRP) proved to be almost equally effective at the short term follow-up (4 and 8 weeks) with slightly better performance by glucocorticoid, while PRP had an upper hand to glucocorticoid in the long term (at 12 weeks) follow-up of the patients (Table IV and V).

**Table IV:** Comparative evaluation of different functional scores with different modalities (Pre and Post infiltration)

	Comparison of VAS Score		
	Normal saline Group	Platelet Rich Plasma Group	Glucocorticoid Group
Pre -treatment	70.6	70.4	70.8
At 4 Weeks	60.8	50.5	50.9
At 8 Weeks	60.6	40.1	50.4
At 12 Weeks	60.2	30.5	40.5
	**Comparison of DASH Score**		
	Normal saline Group	Platelet Rich Plasma Group	Glucocorticoid Group
Pre -treatment	66.8	56.2	55.2
At 4 Weeks	68.6	56.2	42.0
At 8 Weeks	68.0	43.8	35.8
At 12 Weeks	67.8	29.1	32.0
	**Comparison of DASH Score**		
	Normal saline Group	Platelet Rich Plasma Group	Glucocorticoid Group
Pre -treatment	68.8	68.6	67.8
At 4 Weeks	68.6	56.2	62.2
At 8 Weeks	68.0	43.8	54.8
At 12 Weeks	67.8	32.6	46.6
	**Comparison of DASH Score**		
	Normal saline Group	Platelet Rich Plasma Group	Glucocorticoid Group
Pre -treatment	63.4	62.2	63.9
At 4 Weeks	62.8	88.6	78.8
At 8 Weeks	63.6	93.2	88.1
At 12 Weeks	64.0	98.1	88.4

**Table V:** Comparative outcome of Management with Infiltrations of Leucocyte Enriched Platelet-Rich Plasma (LA- PRP), Glucocorticoid and Normal Saline

S. No.	Demographic/ Clinical Characteristics	Normal Saline Group	Activated Platelet Rich Plasma Group	Glucocorticoid Group	P Value
1	**Gender**				
	Male	10	11	09	
	Female	10	09	11	
2	**Mean age**	35.2	34.6	33.8	
3	**Side involved**				
	Right side	12	11	09	
	Left side	08	09	11	
4	**Diabetes mellitus**	01 (Controlled)	01 (Controlled)	00	
5	**Mayo elbow performance score (MEPS) (Average)**				
	Pre-infiltration	63.4	62.2	63.9	
	At 4 weeks	62.8	88.6	78.8	P Value = <0.05
	At 8 weeks	63.6	93.2	88.1	(At 12 Weeks)
	At 12 weeks	64.0	98.1	88.4	
6	**Visual analogue score**				
	Pre-infiltration	70.6	70.4	70.8	
	At 4 weeks	60.8	50.5	50.9	P Value = <0.05
	At 8 weeks	60.6	40.1	50.4	(At 12 weeks)
	At 12 weeks	60.2	30.5	40.5	
7	**DASH score**				
	Pre-infiltration	66.8	56.2	58.2	
	At 4 weeks	68.6	56.2	42.0	P Value = <0.05
	At 8 weeks	68.0	43.8	35.8	(At 12 weeks)
	At 12 weeks	67.8	29.1	32.0	
8	**PRTEE score**				
	Pre-infiltration	63.4	62.2	63.9	
	At 4 weeks	62.8	88.6	78.8	P Value = <0.05
	At 8 weeks	63.6	93.2	88.1	(At 12 weeks)
	At 12 weeks	64.0	98.1	88.4	

Post infiltration increase in the intensity of pain was present in 15 patients, 5 in steroid and 10 in L-aPRP group, which was managed by oral analgesics [(piroxicam 20mg or (etoricoxib 90mg + thiocolchicoside 8mg)] for 3 days.

None of the patients had any sign of infection after the procedure and the results of observations of individual patients were pooled for each intervention group.

Data analysis was performed using SPSS version 20 [SPSS Inc, Chicago, Illinois, USA]. Numerical data were expressed as mean, ± standard deviation (SD) or per cent as proportionate to the sample size. The significance of the difference between the two groups was determined using the "p" value. A "p" value less than 0.05 was considered significant.

## Discussion

Lateral epicondylitis, with an incidence of 1% to 3%, is a familiar chronic disabling painful degenerative condition, occurring at the common origin of the wrist and finger extensors at the elbow due to overuse, and abnormal microvascular responses during post-injury reparative process^4-6^. The basic pathology is in the origin of the extensor carpi radialis brevis (ECRB) tendon, but sometimes the anteromedial edge of the extensor digitorum communis (EDC) and the deep surface of the extensor carpi radialis longus (ECRL) may also be involved^34^. In addition, there is hypervascularity and erratic neovascularisation of the tendon, once injured, leading to erratic revascularisation, defective fibrosis and adhesion, and partial loss of normal function^35,36^. This aberration from normalcy in structure/rearrangement often makes the tissue vulnerable to re-injury^37^.

The injured tendon also develops post-injury interstitial gaps (microtears), discontinuous collagen fibres, degenerative changes like lipid deposition, proteoglycan accumulation, and calcification^38^. It also has a lesser total collagen content, a greater collagen type III/collagen type I ratio, elevated expression of matrix metalloproteinases (MMPs), MM-1, MMP-3, and MMP-9, and decreased expression of the MMP inhibitors^38,39^. Apart from deviations in tendon metabolism, there is intense inflammation at the micro injury site, impairing healing of the tendon tissue if left untreated^40^.

Despite the proliferation of different treatment options for the lateral epicondylitis, reluctance on the part of the patients sways them towards the infiltration therapy either with glucocorticoid or L-aPRP^40-43^. Normal saline infiltrations have a placebo effect for a few days after the infiltration, and the patients present again in the outpatient department with the same complaints.

Corticosteroid injection was the gold standard treatment earlier due to the rapid improvement in signs and symptoms after treatment. However, after a few weeks, there is a recurrence of pain, probably due to the permanent damage of the tendon and hypopigmentation at the infiltration site. Moreover, optimal timing, dosage, injection technique, and injection volume remain unanswered to date.

Autologous PRP was first used to avoid the excessive transfusion of homologous blood products, following open-heart surgery^44^. It is an ideal biological autologous blood-derived component as it is readily available, cost-effective, preventing infection at the infiltration site as it is leucocyte enriched, is without any immune reaction and has potent growth factors required for tendon healing. Leucocyte enriched activated platelets (L-aPRP), when infiltrated, release high concentrations of transforming growth factors, beta (TGF-β), platelet-derived growth factors (PDGF), fibroblast growth factors (FGF), vascular endothelial growth factors (VEGF) and cytokines, through the alfa granules contained within, at the injected site. These growth factors play significant roles in cell proliferation, chemotaxis, cell differentiation, and angiogenesis. In addition, the platelets also secrete several cell adhesion molecules, including fibronectin, fibrin and vitronectin, promoting cell migration and the biological activity of leucocyte enriched platelet-rich plasma (L-aPRP); and promote healing by acting as conductive matrix or scaffold upon which cells can adhere and initiate the healing process^8^.

Decrease in intensity of pain, increase in functional activities, and elbow stability were the main outcome parameters in this study to improve signs and symptoms of lateral epicondylitis. In a study in 2003 to manage lateral epicondylitis, whole blood was injected into patients with a success rate of 79%, but multiple injections were necessary for 32% of patients^4^. Another study in 2006 reported a success rate of 93% with platelet-rich plasma and a 65% success rate with corticosteroids^5^. RP was injected in the elbow of 31 patients in a study in 2011 with failed previous conservative treatment and met the criteria of successful treatment in 90% of patients with a 25% reduction in the worst pain score for at least one follow-up visit, with no further intervention at 12 month interval^14^.

A comparative study in 2011 compared the effectiveness of autologous platelet-rich plasma with steroid therapy in lateral epicondylitis and concluded that platelet-rich plasma injection was safe and easy. Concerning functional impairment, the corticosteroid group showed better results during the initial period and then returned to the baseline. Whereas in the platelet-rich plasma group, symptoms improved progressively and consistently. There was a significant difference in pain and functional impairment after platelet-rich plasma application even after one year. In his study, in the platelet-rich plasma group, the pre-injection DASH score of 54.3 declined to 43.1 at four, 31.2 at 12 weeks. The pre-injection VAS score of 69.0 declined to 55.7 at four weeks, 45.1 at eight and 40.2 at 12 weeks. DASH score among the steroid group declined similarly up to 12 weeks with a decline of VAS score from the pre-injection score of 66.2 to 44.3 at four and 38.5 at 12 weeks^12^.

In the present study, the DASH score among the platelet-rich plasma group declined from a pre-injection score of 56.2, which was the same at four weeks, decreased to 43.8 at eight and 29.1 at 12 weeks. Similarly, the VAS score among the platelet-rich plasma group declined from the pre-injection score of 70.4 to 50.5 at four, 40.1 at eight and 30.5 at 12 weeks. In the present study, the DASH score among the steroid group started to decline from the pre-injection score of 55.2 to 42.0 at four, 35.8 at eight and 34.0 at 12 weeks. In this study, the VAS score among the steroid group declined from 70.8 of pre-injection score to 50.9 at four, 50.4 at eight and 40.5 at 12 weeks.

In another randomised study in 2015, 30 lateral epicondylitis patients, aged 18 - 60 years, with chronic pain (>6 months) were randomised into two groups: group I received a PRP injection and group II received a corticosteroid injection. Patients were assessed using the VAS for pain and Disabilities of the Arm, Shoulder, and Hand (DASH) score. In addition, an ultrasound evaluation of the common extensor origin was performed. At six months, the number of patients positive for various ultrasonographic findings generally decreased. PRP appeared to enable biological healing of the lesion, whereas corticosteroids appeared to provide short-term, symptomatic relief but resulted in tendon degeneration. Improvement in tendon morphology was greater after PRP injection than after corticosteroid injection^45^.

Another randomised-controlled study done in 2013 included 60 patients with lateral epicondylitis divided into three groups. The local injection treatments included a corticosteroid injection of 1ml triamcinolone 40mg/ml+2 ml lidocaine 10mg/ml, a saline injection of 3ml, and 3ml to 3.5ml PRP. All patients were assessed at one and at three months by ultrasonography and PRTEE score. The study found that in terms of PRTEE at one month, corticosteroid was superior to both PRP and saline, but at three months, its effect declined^46^.

A study in 2015 carried out on 65 patients with lateral epicondylitis, divided them randomly into two groups: group A received a single infiltration of one ml PRP with an absolute platelet count of at least one million platelets / mm3, and group B had a single injection of one ml (40mg) methylprednisolone. VAS was used to assess post infiltration pain. It had greater improvement with a corticosteroid injection after 15 days and one month than with PRP; however, it declined, and at the end of three months, improvement in pain was highly significant in the PRP group compared to the corticosteroid group (P<0.0001)47. The superior effect of corticosteroid early during treatment might be because of its anti-inflammatory effect, whereas the late positive effect was noted in the PRP group over the corticosteroid effect. Nevertheless, it was in agreement with our study and is because of the higher healing power of the PRP over the corticosteroid.

A treatment protocol in 2017 included 45 patients with LE (more than three months) between 31 and 58 years of age. The patients were divided randomly into three groups: group I received a saline injection, group II received a PRP injection, and group III received a corticosteroid injection. Patients were reassessed clinically and by ultrasound after three months. They showed that VAS and PRTEE scores were significantly reduced after injection in group II compared to group I and III. Moreover, the reductions in VAS and PRTEE were significantly different in group III in comparison with group I48. With the results of the 12 weeks follow-up, the outcome in the platelet-rich plasma group was maintained, whereas the outcome in the corticosteroid group declined; and significantly, the platelet-rich plasma group which had poorer pre-injection VAS scores but better scores after 12 weeks.

This strengthens the view that the platelet-rich plasma is undoubtedly a better alternative to corticosteroid in lateral epicondylitis. However, the limitation of our study is the very small sample size, and a larger database will be needed to confirm its findings.

## Conclusion

L-aPRP is more beneficial therapeutically than corticosteroid infiltration as it is cost-effective and readily available. It contains growth factors for healing, and being an autologous preparation, it is immunologically compatible and has antibacterial activity from enrichment with the leucocytes. Moreover, it has a continuous, longer duration of action. It enables better healing as it leads to a more homogenous tendon arrangement and systematic neovascular proliferation post-injury in occupational and sports injuries. Normal saline injections are just placebo injections and have no role in the management of lateral epicondylitis.
